# Women’s reproductive health knowledge, attitudes and practices in relation to the Zika virus outbreak in northeast Brazil

**DOI:** 10.1371/journal.pone.0190024

**Published:** 2018-01-03

**Authors:** Ana Luiza Vilela Borges, Caroline Moreau, Anne Burke, Osmara Alves dos Santos, Christiane Borges Chofakian

**Affiliations:** 1 Department of Public Health Nursing, School of Nursing, University of São Paulo, São Paulo, São Paulo, Brazil; 2 Department of Population, Family, and Reproductive Health, Bloomberg School of Public Health, The Johns Hopkins University, Baltimore, Maryland, United States of America; 3 Department of Gynecology and Obstetrics, Johns Hopkins Bayview Medical Center, Johns Hopkins University School of Medicine, Baltimore, Maryland, United States of America; 4 Gender, Sexual and Reproductive Health, CESP Centre for Research in Epidemiology and Population Health, U1018, Inserm, Le Kremlin-Bicêtre, France; TNO, NETHERLANDS

## Abstract

**Objectives:**

To assess knowledge, pregnancy attitudes and contraceptive practices in relation to the Zika virus outbreak in Brazil.

**Methods:**

We interviewed 526 women 18 to 49 years old in primary health services in a Northeastern capital of Brazil, in 2016. They provided information about their knowledge of Zika transmission and health consequences, their receipt of counseling related to sexual and perinatal transmission of Zika, their pregnancy intentions and reassessment of contraceptive options in the context of the Zika virus outbreak.

**Results:**

Awareness about Zika congenital syndrome was high, but knowledge about sexual transmission was low. Few women had changed pregnancy intentions or contraceptive practices in response to Zika. Pregnant women were more likely to access counseling about family planning, condom use and pregnancy postponement due to Zika virus than non-pregnant women, which may suggest that health system responses followed pregnancy occurrence.

**Conclusions:**

We observed missed opportunities for prevention of perinatal transmission of Zika virus through behavioral change, including effective contraception to prevent pregnancy and condoms to prevent perinatal transmission, as a complement to vector control.

## Introduction

The Zika virus outbreak is an unprecedented health emergency, affecting 70 countries and territories since 2015 [1]. Brazil is the epicenter of this outbreak. The first autochthonous Zika transmitted cases were documented in April 2015, followed five months later by the first cases of Zika related congenital syndrome. Since then, more than 200,000 Zika cases and 2,753 cases of Zika congenital syndrome were confirmed in Brazil until May 2017 [2,3].

Brazil’s public health response to the Zika outbreak has mostly focused on epidemiological surveillance and vector control. Past efforts to control Dengue and Chikungunya—other arboviral diseases transmitted by the same mosquito, *Aedes egypti*—prove this strategy challenging, highlighting the need for a multi-sectoral approach. In particular, the unique consequences of Zika on maternal and perinatal health call for a broader spectrum of public health interventions, including primary prevention to reduce incidence of unwanted pregnancies. Whereas many Latin American countries have officially advised women to avoid becoming pregnant in Zika affected areas, the Brazilian Ministry of Health has only stressed the importance of counseling and access to family planning. The Brazilian guidelines for addressing Zika-related microcephaly, published in March 2016 [4], acknowledge the role of contraception but fail to address barriers to access effective contraceptive methods, including long acting reversible contraception (LARC) [5].

While contraceptive prevalence is high in Brazil (77.7% of modern method use among married women), 46% of pregnancies were unintended according to the last Demographic and Health Survey conducted in 2006 [6]. This high proportion of unintended pregnancies reveals the shortcomings of the Brazilian contraceptive model, which mainly focuses on female sterilization for limiting childbearing or on short-acting user-dependent methods for delaying or spacing childbearing (pills, injectables and condoms). Short acting methods, which have high discontinuation and failure rates, are the most popular methods for women at the peak of their fertile years [6,7]. While there are no recent data tracking trends in family planning indicators in Brazil, there is little reason to believe the country has significantly improved its family planning landscape in the last 10 years. Indeed, surveys conducted among specific populations in Brazil point to the contrary [8, 9], suggesting multilevel barriers to the uptake of more effective methods, such as LARC, from policies to programs, to women’s decision making. Such barriers include unavailability of certain methods (implants and hormonal IUD are not available in Brazilian public health services), stock outs, and lack of trained professionals [9, 10]. The lack of efficiency of the Brazilian contraceptive model has particular resonance in the face of restricted abortion policies, with unsafe terminations contributing 12% of maternal mortality in Brazil [11].

As nothing indicates the country will soon reconsider its position on abortion, suboptimal contraceptive services are likely to be of particular consequence for maternal and perinatal health in the country and especially in the Northeast region, most severely affected by Zika virus transmission.

As Brazil transitions from the second to third year of a Zika virus outbreak, this study aims to provide new insights on women’s knowledge of Zika-related sexual and reproductive health concerns, their receipt of counseling related to sexual and perinatal transmission of Zika, their pregnancy intentions in the context of the Zika outbreak, and their reassessment of contraceptive options in light of Zika concerns. The study focuses on women living in the Northeast region.

## Materials and methods

This study is drawn from a larger cross-sectional project “Patterns and determinants of contraceptive discontinuation in Brazil” evaluating the frequency and correlates of contraceptive discontinuation among women attending primary health care facilities in three cities across different regions in Brazil. Data collection in the Northeast Region took place in August and September 2016 in the city of Aracaju, capital of Sergipe, located between the states of Bahia and Alagoas, whose border is with Pernambuco and Paraíba, where the recent congenital syndrome associated with Zika virus was first observed. In 2016, the Zika incidence rate in Sergipe was estimated at 4.68 cases per 100,000, and 128 cases of Zika related microcephaly were confirmed in the State with 58 additional cases under investigation [12].

A two-stage probability sampling procedure was used to select primary health care facilities at the first stage and women attending the services at the second stage. Out of 42 primary health care facilities in the city, 19 were selected as a function of proportional caseload based on the number of Pap smear tests completed in 2015. Primary health care facilities participating in this study are part of the Brazilian Health System (Sistema Único de Saúde/SUS), which provides universal preventive and curative care to over 200 million people. All these facilities provide a range of women’s health services including medical and nursing consultations, prenatal care, family planning, immunization, Pap smear tests and breast cancer screening.

From each facility, we selected a convenience sample of women aged 18 to 49 years. The first 27 women waiting for medical or nursing consultation during the three days of fieldwork in each facility were invited to participate in the study. Exclusion criteria were: 1) not having initiated sexual activity and 2) had been sterilized for more than 5 years. This last criterion was related to the main objective of the larger project assessing contraceptive discontinuation.

A total of 1005 women with scheduled medical or nursing consultations were approached in the waiting rooms of the facilities; 974 (97%) chose to participate and signed an informed consent before the interview started. Some women did not meet eligibility criteria, as they had never had sexual intercourse, were sterilized for more than 5 years or were not in the age range of the study (18–49) (n = 448). Altogether 526 women were interviewed by trained nurses or psychologists in private offices. The interviewers entered the data using tablets equipped with Census and Survey Processing System software (CSPro). A random sample of 10% of questionnaires was taken from Stata 14.2 and checked for quality purposes by re-interviewing participants by telephone. We observed high consistency between the answers to the face-to-face interview and the telephone interview. The ethics board of the School of Nursing at University of São Paulo approved the study.

The 20-minute questionnaire included questions on women’s socio-demographic characteristics, sexual and reproductive histories, contraception use at last intercourse, and pregnancy history. Variables considered were age, paid work, education, social status (based on the Brazilian Economic Classification Criteria, which uses a methodology to classify households based on purchasing power of household appliances, householder`s education, and family income) [13], marital status, private health insurance, number of births, future pregnancy intention, contraceptive use at last sexual intercourse (none or less effective, including barrier/natural methods and more effective, including hormonal and intrauterine methods), switched to more effective method since Zika outbreak, and used emergency contraception since Zika outbreak.

Specific questions were introduced to assess women’s knowledge and attitudes regarding Zika. An example of the questionnaire used to interview women is available at S1 File. Women were asked if they had already heard of Zika virus. The six women who had not heard of Zika virus were excluded from the present analysis that focuses on reproductive attitudes related to Zika. Thus our analytical sample comprises 520 women. These women were asked about knowledge of sexual transmission of Zika, awareness of Zika related microcephaly, and receipt of counseling about pregnancy postponement in relation to Zika. Non-pregnant women were also asked if they had recently changed their pregnancy intentions in relation to Zika and if they had reevaluated their contraceptive choices because of the outbreak. Pregnant women were asked if the pregnancy was intended and if they were using condoms to prevent Zika transmission during pregnancy.

We used descriptive statistics to explore women’s knowledge and attitudes regarding Zika-related sexual and reproductive health concerns, their receipt of counseling related to Zika according to their socio-demographic characteristics, their parity and their current contraceptive practice. Multivariate logistic regression was conducted to assess variables associated with changing pregnancy intention in relation to Zika virus outbreak.

## Results

The description of the sample is provided in Table 1. On average, women were 30.5 years old (sd = 8.0), and 70.6% were married. Women reported on average 9.2 years of schooling (sd = 3.1), 92.5% did not have private health insurance and 42.0% received a social benefit such as the Bolsa Familia Programe, which is a national conditional cash transfer program for poor families (the rest received no social benefit).

**Table 1:** Sociodemographic and reproductive characteristics of women in the sample.

Variable	n	%
**Age (in years)**		
18–24	143	27.5
25–34	219	42.1
35–49	158	30.4
**Educational level (in years)**		
0–8	193	37.1
9–11	287	55.2
>11	40	7.7
**Social status**		
High	43	8.3
Middle	285	54.8
Low	192	36.9
Work paid job*		
No	256	49.4
Yes	263	50.6
**Private health insurance**		
No	481	92.5
Yes	39	7.5
**Married**		
No	153	29.5
Yes	367	70.6
**Current pregnancy status**		
Pregnant	123	29.4
Non-pregnant	397	70.6
**Number of births**		
0	125	24.0
1	184	35.4
>1	211	40.6
**Future pregnancy intention**		
Wants (more) children	157	30.2
Does not want to have (more) children	338	65.0
Not sure	25	4.8
Contraceptive use at time of survey**		
No	87	21.9
Yes	310	78.1
**Switched to more effective method since Zika outbreak**		
No	514	98.8
Yes	6	1.2
**Used emergency contraception since Zika outbreak**		
No	434	83.5
Yes	86	16.5
**Total**	**520**	**100**

* 1 woman failed to answer

**123 pregnant women not considered

Most women (65%) reported no intention to have (more) children in the future. A quarter of women (n = 123) were pregnant at the time of the survey, with 59.9% of these pregnancies described as unintended. A minority of women (7.1%) was trying to get pregnant at the time of the survey, while 66.5% (n = 346) were at potential risk of unintended pregnancy (not pregnant, not trying to conceive, not infertile and sexually active). Among the 346 women at risk, 2% were using a highly effective method (IUD), 50.9% were using moderately effective methods (29.5% pills and 21.4% injectables), and 24.9% were using less effective methods (21.1% condoms and 3.8% traditional methods). Finally, 22.2% of women at risk were using no method (unmet need).

### Knowledge about Zika

Almost every woman who had ever heard about Zika (n = 520) was aware of Zika virus’ relation to congenital syndrome (98.6%) but only half was aware that Zika virus could be transmitted by sexual relations (50.2%). Table 2 shows that the knowledge of sexual transmission did not vary by age, parity or social background, although the association with educational level was close to significance, with increasing knowledge with increasing years of schooling (p = 0.063). Pregnant women who were using condoms at the time of the survey were more likely to be aware of Zika sexual transmission (p = 0.002) while this association was not significant among non-pregnant women. Among pregnant women, 10.6% were using condoms to prevent Zika transmission during pregnancy.

**Table 2:** Awareness that Zika virus can be transmitted sexually.

Total	All (n = 520) % aware of Zika sexual transmission	p	Non pregnant (n = 397) % aware of Zika sexual transmission	p	Pregnant (n = 123) % aware of Zika sexual transmission	p
	50.2% (n = 261)		50.4% (n = 200)		49.6% (n = 61)	
**Age (in years)**		0.704		0.292		0.695
18–24	53.2		57.4		44.9	
25–34	49.3		48.1		52.5	
35–49	48.7		48.3		53.8	
**Educational level (in years**)		0.063		0.116		0.547
0–8	47.2		47.3		46.7	
9–11	49.8		50.0		49.3	
>11	67.5		67.7		66.7	
**Social status**		0.074		0.241		0.162
High	51.2		54.3		41.2	
Middle	54.4		53.4		57.8	
Low	43.7		44.7		37.5	
**Number of births**		0.105		0.092		0.777
0	58.4		61.2		53.3	
1	48.4		48.2		48.9	
>1	46.9		47.2		45.2	
**Condom use**^1^		0.006		0.111		0.002
No	47.4		48.4		44.4	
Yes	63.3		58.7		86.7	

^1^Condom use for any reason among non-pregnant women; for pregnant women, this was a specific question regarding condom use due to Zika outbreak

### Counseling about family planning, condom and pregnancy postponement

Only 9.3% of non-sterilized women were asked about their pregnancy intentions by their healthcare provider during the Zika virus outbreak, 11.6% were advised to avoid pregnancy and 14.4% were counseled about condoms to avoid sexual transmission of Zika virus (Table 3). The majority of women received none of these recommendations (78.2%), while 18.6% received one or two and 3.2% received the three types of information.

**Table 3:** Discussion about pregnancy intentions and counseling about condom use in relation to the Zika epidemic.

	Health professional asked about reproductive intention during Zika virus outbreak	p	Health professional advised to avoid becoming pregnant because of Zika virus outbreak	p	Health professional recommended the use of condoms to avoid Zika virus	p
Total (non sterilized women) (n = 506)	9.3% (n = 47)		11.6% (n = 59)		14.4% (n = 73)	
**Age**		0.314		0.013		0.585
18–24	9.1		13.3		16.8	
25–34	11.3		15.1		14.1	
35–49	6.6		5.3		12.6	
**Parity**		0.072		0.362		0.927
0	14.4		15.2		15.2	
1	8.2		10.3		14.7	
>1	7.1		10.7		13.7	
**Pregnant**		0.007		0.013		0.015
No	7.3		9.7		12.3	
Yes	15.4		17.9		21.1	
**Social status**		0.196		0.594		0.398
High	16.3		16.3		12.2	
Middle	9.4		10.9		16.4	
Low	7.5		11.7		11.6	
**Educational level**		0.144		0.318		0.706
0–8	9.6		12.8		14.9	
9–11	7.9		10.0		14.7	
>11	17.5		17.5		10.0	
**Contraception**		0.088		0.067		0.085
No	11.9		14.8		17.6	
Yes	7.4		9.5		12.2	
**Switched to more effective methods since Zika outbreak**		0.277		0.404		0.546
Yes	9.2		11.5		14.3	
Yes	25.0		25.0		25.0	
**Used emergency contraception since Zika outbreak**		0.042		0.455		0.592
No	10.5		12.1		14.0	
Yes	3.5		9.3		16.3	

The most consistent predictor of Zika virus-related reproductive counseling was pregnancy, as pregnant women were more likely to indicate having discussed pregnancy intentions, pregnancy delay and condom use to prevent Zika transmission. Contraceptive use at the time of the survey was also marginally related to health provider’s advice about avoiding pregnancy (p = 0.067), while women using EC were less likely to talk about their pregnancy intentions with a healthcare provider during the Zika virus outbreak (p = 0.042). Women under 35 years of age were more likely to be advised to avoid pregnancy because of Zika (p = 0.013). The percentage of women who reported receiving counseling did not vary by women’s social and educational background.

### Attitudes towards pregnancy and contraception as related to Zika virus outbreak among women at risk

Pregnancy intentions and attitudes towards contraception in relation to the Zika outbreak among women who were not pregnant at the time of the survey are reported in Table 4. Only 12.4% of these women indicated that Zika virus outbreak influenced their pregnancy intentions. The survey did not query details about this change in intention. Change in pregnancy intention was associated with age (p = 0.003), parity (p = 0.007) and future reproductive intention (p<0.001), with women over 35, women at parity 2 or more and women who were sure about having (more) children, less likely to indicate Zika had influenced their pregnancy intentions (all these significant p-values are from unadjusted analyses). Conversely, women using more effective methods (hormonal methods or IUD) were more likely to indicate that they had changed their pregnancy intentions in response to Zika. While change in pregnancy intentions was not related to educational level, it was marginally related to social status (p = 0.079). Only future pregnancy intention remained significantly related to changes in pregnancy intentions in the multivariate analysis.

**Table 4:** Change in pregnancy intentions related to the Zika virus among women at risk.

	Women at risk (n = 346)		
	% who changed pregnancy intention in relation to Zika	p*	OR adjusted changed pregnancy intention in relation to Zika
Total	12.4 (n = 43)		
**Age**		0.003	
18–24	18.3		1
25–34	16.4		1.34 (0.58–3.09)
35–49	4.6		0.40 (0.12–1.29)
**Parity**		0.007	
0	18.2		1
1	17.2		1.25 (0.48–3.20)
>1	6.3		0.48 (0.14–1.61)
**Social status**		0.079	
High	24.2		3.04 (0.91–10.21)
Middle	10.2		0.90 (0.39–2.07)
Low	12.6		1
**Educational level (years)**		0.979	
0–8	12.8		1
9–11	12.1		0.49 (0.21–1.14)
>11	13.0		0.31 (0.06–1.66)
**Contraception use**		0.041	
None or less effective	8.6		1
More effective	15.8		0.52 (0.25–1.09)
**Future pregnancy intention**		<0.001	
Wants (more) children	19.1		1
Does not want to have (more) children	7.7		0.55 (0.23–1.34)
Not sure	36.4		4.00 (1.27–12.47)
**Switched to more effective method since Zika outbreak**			
No	100.0		
Yes	-		
**Used emergency contraception since Zika outbreak**		0.730	
No	12.1		1
Yes	13.8		0.97 (0.39–2.40)

* Unadjusted, bivariate analysis

Only five women at risk (1.4%) indicated that the Zika virus outbreak had influenced their contraception choice, precluding the analysis of Zika virus outbreak influence in contraceptive decisions.

## Discussion

This is the first study to provide information about women’s knowledge and reproductive health attitudes and practices in relation to Zika virus. Our results show that awareness about Zika virus related to congenital syndrome is high, but that knowledge about sexual transmission is low. These results reflect missed opportunities for prevention of perinatal transmission of Zika through behavioral change (contraception to prevent pregnancy and condoms to prevent perinatal transmission once a woman is pregnant) as a complement to vector control. Only one out of ten pregnant women was using condoms to prevent sexual transmission of Zika during pregnancy, with an unsurprising higher usage among those who were aware of sexual transmission risk. Those findings highlight the urgency of improving access to consistent and timely information among pregnant women in Brazil [14].

Our findings also show sparse reproductive health counseling related to the Zika outbreak. Most women were not asked about their reproductive intentions, were not advised about contraception to delay pregnancy and were not informed about condom use to prevent perinatal transmission. These results are concerning, especially as women in the study were attending public health services for medical consultations and all primary health professional teams working in the city of Aracaju had received training about Zika virus (Borges CL, Aracaju Health Office, Personal Communication, September 2016). Our findings suggest that training, which probably focused on diagnosis and clinical management of Zika over prevention, should extend to incorporate family planning preventive measures.

Few women reported being advised to avoid pregnancy because of the Zika virus outbreak, this proportion increasing among younger women and pregnant women. As delaying pregnancy is not part of the Brazilian guidelines for addressing Zika-related microcephaly, some health professionals may have taken the initiative to counsel women in response to the national debate about reproductive services and outcomes related to the Zika threat, including abortion restrictions and perinatal anomalies.

Pregnant women were more likely to access counseling about family planning, condom and pregnancy postponement due to Zika virus than non-pregnant women, which may suggest that health responses followed pregnancy occurrence. The lack of emphasis on primary prevention of pregnancy through family planning is especially concerning in view of the 60% of pregnancies that were deemed unintended in our study population. Another indicator of inadequate family planning response to Zika in the Northeast region is the missed opportunity to counsel women about contraception when they use emergency contraception. In Brazil, emergency contraception is mostly accessed in private pharmacies. Poor emergency contraception counseling from pharmacists in the country [15] may explain why women who use it received no contraceptive counseling about family planning in relation to Zika. The low level of information found among pregnant women who had more opportunities to interact with health professionals working in public health facilities through prenatal care (Brazil presents high coverage of prenatal care) [16] suggests shortcomings in reproductive health counseling across a wide range of primary care settings, reducing women’s opportunity to prevent Zika-related perinatal morbidity.

Conversely to a Brazilian national study conducted in June 2016 where 56% of women reported they had avoided (or tried to avoid) pregnancy because of Zika epidemic [17], our findings show that Zika virus outbreak seems to have not largely influenced women`s reproductive intention in Aracaju, Sergipe, with just one out of eight women having reported pregnancy intention change since then. Women who were not sure about future pregnancy intention were more likely to change their pregnancy intentions in relation to Zika virus than women who were sure about their intentions, whether they wanted more or no more children. As expected, women who did not want to have (more) children (the majority in our sample), so-called limiters, were not likely to change their pregnancy intentions irrespective of the Zika outbreak. From the perspective of women who are determined to have (more) children, uncertainty about if and when the Zika epidemic will recede or end in Brazil may deter them from delaying their reproductive plans, especially given the absence of success in controlling other endemic mosquito transmitted diseases such as dengue. Asking all women about their pregnancy intentions should be part of routine care in order to address their needs both for contraception and preconception care [18], especially in a context of Zika virus outbreak.

Few women in our sample were using LARCs, which reflects Brazil’s contraceptive model focused on short acting methods. Because short acting methods are more likely to fail and be discontinued [19, 20], these findings highlight the missed opportunity for public health services to improve contraceptive efficacy for women who wish to avoid pregnancy, which constitutes the majority of the women in this sample. Those results also question the national strategy calling for increases in contraceptive supplies to reduce Zika congenital disease but fail to mention LARCs, which is almost nonexistent in Brazil [4]. Since the beginning of the outbreak, there has been no significant change in contraceptive sales in Brazil [21]. In the absence of specific programs promoting LARCs, it is unlikely that method mix has changed, with limited use of the most effective methods as suggested in our study. Given the frequency of unintended pregnancies among women attending public primary care settings, there is urgent need for public sector providers to counsel women about contraception as a primary prevention for unintended pregnancy and the associated risk of Zika in order to reduce the high levels of unmet need observed in our study and offer a wide range of effective contraceptive options to improve prevention. The Zika virus outbreak is an opportunity to improve women’s health by strengthening family planning services. This is especially important in a country where abortion is restricted, as the Zika virus outbreak may influence abortion decision-making[5]. Aiken et al. (2016) [22] observed an increase in online requests for abortion medications in countries with autochthonous Zika virus transmission and abortion restriction, Brazil included. This is a concern since unsafe abortions are linked to maternal morbidity and mortality rates [23].

This study is one of the first to evaluate reproductive health counseling and women’s childbearing and contraceptive decision making in relation to Zika in Brazil, in one of the most affected regions. The study has a number of limitations. A first concern relates to the seasonality of Zika outbreak in Brazil: as women were interviewed in winter in Brazil, at a time of low mosquito infestation and, consequently, Zika virus transmission [24], we may have underestimated the effect of Zika on women’s decision making. Had the survey been conducted in the summer, at the height of Zika virus incidence, it is possible that our findings about contraceptive practices and decision-making may have differed. This may partially explain differences seen with the recent national study [17]; although the study populations were also different, with our sample targeting less advantaged women consulting in public sector primary care services. Other limitations relate to fact that we collected no information on whether women had been previously diagnosed with Zika virus. Finally, the cross-sectional nature of the study renders some findings difficult to interpret (more frequent discussions about pregnancy prevention among pregnant than non-pregnant women for instance), as well as the lack of follow up questions asking details about provision of reproductive counseling and the nature of the information that was shared.

## Conclusions

Women of childbearing age in Northeastern Brazil are vulnerable to Zika virus perinatal transmission. The reason is that they are not fully aware that Zika virus can be sexually transmitted and they have poor access to counseling about how to prevent Zika virus transmission, even during a pregnancy. Pregnancy intention has not changed due to the outbreak, except for women who are not sure about having (more) children. Reproductive response to the Zika virus outbreak needs to expand correct and timing information so women can be able to plan their pregnancy and pregnant women can prevent perinatal transmission.

## Supporting information

S1 File Questionnaire used to interview women—English and Portuguese version. (PDF)
