# Correction: Women’s reproductive health knowledge, attitudes and practices in relation to the Zika virus outbreak in northeast Brazil

**DOI:** 10.1371/journal.pone.0195150

**Published:** 2018-03-26

**Authors:** The PLOS ONE Staff

There are errors in the author affiliations. The publisher apologizes for these errors. The affiliations should appear as shown here: Ana Luiza Vilela Borges^1^*, Caroline Moreau^2,4^, Anne Burke^2,3^, Osmara Alves dos Santos^1^, Christiane Borges Chofakian^1^

**1** Department of Public Health Nursing, School of Nursing, University of São Paulo, São Paulo, São Paulo, Brazil, **2** Department of Population, Family, and Reproductive Health, Bloomberg School of Public Health, The Johns Hopkins University, Baltimore, Maryland, United States of America, **3** Department of Gynecology and Obstetrics, Johns Hopkins Bayview Medical Center, Johns Hopkins University School of Medicine, Baltimore, Maryland, United States of America, **4** Gender, Sexual and Reproductive Health, CESP Centre for Research in Epidemiology and Population Health, U1018, Inserm, Le Kremlin-Bicêtre, France.
