# Supplementary material for: Women’s reproductive health knowledge, attitudes and practices in relation to the Zika virus outbreak in northeast Brazil
Source: PLoS One. 2018 Jan 3;13(1):e0190024. doi: 10.1371/journal.pone.0190024 (PMC5752017; doi:10.1371/journal.pone.0190024)
Supplement: S1 File — (PDF) [file pone.0190024.s001.pdf]

Women's Reproductive Health Knowledge, Attitudes and Practices in Relation to the Zika Virus Outbreak in Northeast Brazil

Dr Ana Luiza Borges
School of Nursing
University of Sao Paulo
Brazil

Questionário (original in Portuguese)

Identificação do questionário:
Número do questionário: __ __ __ __
Data: __/__/__
UBS: [fechada]

Características sociodemográficas:
1. Qual é a sua data de nascimento? ___/___/___ [preencher em dia/mês/ano]

2. Qual foi a última série que concluiu com aprovação? __ __ anos de escolaridade

3. Como você classifica sua cor? [estimulada]
    1 ( ) Branca
    2 ( ) Parda
    3 ( ) Preta
    4 ( ) Amarela
    5 ( ) Indígena

4. Você exerce alguma atividade remunerada?
    0 ( ) Não
    1 ( ) Sim

5. Está procurando emprego atualmente?
    0 ( ) Não
    1 ( ) Sim

6. Você tem plano de saúde ou convênio?
    0 ( ) Não
    1 ( ) Sim

7. Atualmente está casada ou em união com alguém? [estimulada]
    0 ( ) Não
    1 ( ) Sim

Características socioeconômicas:

8. Na residência em que você mora, há quantos banheiros?
    0 ( ) Nenhum
    1 ( ) 1
    2 ( ) 2
    3 ( ) 3

4 ( ) 4 ou mais

9. Na residência em que você mora, há quantas(os) empregadas(os) domésticas(os) mensalistas, quer dizer, que trabalham em sua casa de modo permanente por cinco ou mais dias por semana, incluindo babás, motoristas, cozinheiras, etc?
0 ( ) Nenhum(a)
1 ( ) 1
2 ( ) 2
3 ( ) 3
4 ( ) 4 ou mais

10. Na residência em que você mora, há quantos automóveis/carro para uso pessoal ou da família (não considerar taxis, vans ou caminhonetes usadas para fretes, ou qualquer veículo usado para atividade profissional)?
0 ( ) Nenhum
1 ( ) 1
2 ( ) 2
3 ( ) 3
4 ( ) 4 ou mais

11. Na residência em que você mora, há quantos microcomputadores?
0 ( ) Nenhum
1 ( ) 1
2 ( ) 2
3 ( ) 3
4 ( ) 4 ou mais

12. Na residência em que você mora, há quantas máquinas de lavar louças?
0 ( ) Nenhuma
1 ( ) 1
2 ( ) 2
3 ( ) 3
4 ( ) 4 ou mais

13. Na residência em que você mora, há quantas geladeiras?
0 ( ) Nenhuma
1 ( ) 1
2 ( ) 2
3 ( ) 3
4 ( ) 4 ou mais

14. Na residência em que você mora, há quantos freezers? (considerar aparelho independente ou 2ª porta externa da geladeira duplex)
0 ( ) Nenhum
1 ( ) 1
2 ( ) 2
3 ( ) 3
4 ( ) 4 ou mais

15. Na residência em que você mora, há quantas máquinas de lavar roupa?
0 ( ) Nenhuma
1 ( ) 1
2 ( ) 2
3 ( ) 3
4 ( ) 4 ou mais

16. Na residência em que você mora, há quantos aparelhos de DVD?
    0 ( ) Nenhum
    1 ( ) 1
    2 ( ) 2
    3 ( ) 3
    4 ( ) 4 ou mais

17. Na residência em que você mora, há quantos aparelhos de microondas?
    0 ( ) Nenhum
    1 ( ) 1
    2 ( ) 2
    3 ( ) 3
    4 ( ) 4 ou mais

18. Na residência em que você mora, há quantas motocicletas?
    0 ( ) Nenhuma
    1 ( ) 1
    2 ( ) 2
    3 ( ) 3
    4 ( ) 4 ou mais

19. Na residência em que você mora, há quantas máquinas de secar roupa?
    0 ( ) Nenhuma
    1 ( ) 1
    2 ( ) 2
    3 ( ) 3
    4 ( ) 4 ou mais

20. Qual é a escolaridade do chefe de sua família?
    0 ( ) Analfabeto/ Fundamental 1 Incompleto
    1 ( ) Fundamental 1 Completo/ Fundamental 2 Incompleto
    2 ( ) Fundamental 2 Completo/ Médio Incompleto
    3 ( ) Médio Completo/ Superior Incompleto
    4 ( ) Superior Completo

21. Você ou alguém do seu domicílio recebe algum tipo de benefício social?
    0 ( ) Não
    1 ( ) Seguro-desemprego
    2 ( ) Bolsa-família
    3 ( ) Bolsa-escola
    4 ( ) Pensão
    5 ( ) Aposentadoria
    6 ( ) outro

22. Na rua em que você mora, tem água encanada?
    0 ( ) Não
    4 ( ) Sim

23. A rua em que você mora é pavimentada?
    0 ( ) Não
    2 ( ) Sim

Agora necessitamos de algumas informações sobre sua atividade sexual e história reprodutiva, para melhor entender as questões de saúde reprodutiva.

24. Que idade tinha quando teve a primeira relação sexual? __ __

25. Quantos parceiros sexuais teve na vida? __ __

26. Está grávida atualmente?
- 0 ( ) Não
- 1 ( ) Sim [pular para questão 28]
- 2 ( ) Não sei

27. Já engravidou alguma vez (considerar mesmo que a gravidez não tenha chegado ao fim)?
- 0 ( ) Não [pular para questão 31]
- 1 ( ) Sim

28. Quantas vezes já engravidou?__ __

29. Já teve algum abortamento?
- 0 ( ) Não
- 1 ( ) Sim

30. Quantos filhos vivos tem atualmente? __ __

31. Qual é a sua intenção der ter filhos? [estimulada]
- 1 ( ) Quer ter filhos nos próximos dois anos
- 2 ( ) Quer ter filhos no futuro
- 3 ( ) Não quer ter (mais) filhos
- 4 ( ) Não sabe

Contracepção:

32. Atualmente, está usando um método contraceptivo para evitar gravidez?
- 0 ( ) Não [Pular para questão 35]
- 1 ( ) Sim

33. Qual é o método contraceptivo que está usando atualmente? [marcar o mais eficaz] Estimulada. Marcar apenas um método mais eficaz
- 1 ( ) Pílula oral
- 2 ( ) Hormonal injetável
- 3 ( ) Preservativo masculino
- 4 ( ) Preservativo feminino
- 5 ( ) DIU
- 6 ( ) Diafragma
- 7 ( ) Tabelinha
- 8 ( ) Coito interrompido
- 9 ( ) Pílula do dia seguinte
- 10 ( ) Implante
- 11 ( ) Adesivo
- 12 ( ) Anel vaginal
- 13 ( ) Laqueadura [há mais de 5 anos? Se sim, pesquisa termina aqui]
- 14 ( ) Vasectomia [há mais de 5 anos? Se sim, pesquisa termina aqui]
- 15 ( ) Outro – Cite qual __________________.

34. [caso tenha citado mais de um método contraceptivo, marque o método a seguir] [resposta única]

1 ( ) Pílula oral
2 ( ) Hormonal injetável
3 ( ) Preservativo masculino
4 ( ) Preservativo feminino
5 ( ) DIU
6 ( ) Diafragma
7 ( ) Tabelinha
8 ( ) Coito interrompido
9 ( ) Pílula do dia seguinte
10 ( ) Implante
11 ( ) Adesivo
12 ( ) Anel vaginal
10 ( ) Outro – Cite qual__________________.

Agora vamos perguntar sobre a contracepção de emergência, também chamada de pílula do dia seguinte.

35. Você já usou a contracepção de emergência?
0 ( ) Não [pular para questão 40]
1 ( ) Sim

36. Quantas vezes já usou a contracepção de emergência na vida? __ __

37. No último ano, quantas vezes usou a contracepção de emergência? __ __

38. Da última vez em que usou a contracepção de emergência, você estava usando algum outro método contraceptivo?
0 ( ) Não [pular para questão 39]
1 ( ) Sim

58-1. Se sim, qual método? [Anotar o mais eficaz] (Resposta única)
1 ( ) Pílula oral
2 ( ) Hormonal injetável
3 ( ) Preservativo masculino
4 ( ) Preservativo feminino
5 ( ) DIU
6 ( ) Diafragma
7 ( ) Tabelinha
8 ( ) Coito interrompido
9 ( ) Implante
10 ( ) Adesivo
11 ( ) Anel vaginal
12 ( ) Outros – Cite qual ou quais___________.

39. Da última vez em que usou a contracepção de emergência, você foi orientada por um profissional de saúde sobre o método?
0 ( ) Não
1 ( ) Sim

Agora vamos perguntar sobre o virus Zika

40. Você já ouviu falar sobre o vírus Zika?
0 ( ) Não
1 ( ) Sim

41. Você já ouviu falar que o Vírus da Zika está relacionado com os casos de microcefalia nos bebês?

0 ( ) Não

1 ( ) Sim

No final de 2015, teve início um surto do vírus Zika, que persiste até hoje, com casos de microcefalia notificados em todo o Brasil. Por conta disso, algumas mulheres e casais decidiram adiar a gravidez. Outros acham que isso não é necessário. E quanto a você?

42. Você acha que o surto do virus Zika está influenciando seus planos de engravidar?

0 ( ) Não

1 ( ) Sim

43. Quando gostaria de engravidar?

0 ( ) Imediatamente [pular para questão 46]

1 ( ) Daqui algum tempo [pular para questão 44]

2 ( ) Nunca (mais) [pular para questão 46]

3 ( ) Não tem certeza [pular para questão 46]

44. Você acha que o surto do vírus Zika influenciou sua intenção de engravidar somente daqui algum tempo?

0 ( ) Não [pular para questão 46]

1 ( ) Sim [pular para questão 46]

45. Você acha que o surto do vírus Zika influenciou o fato de não ter certeza sobre sua intenção de não engravidar?

0 ( ) Não

1 ( ) Sim

46. Algum profissional de saúde perguntou sobre sua intenção em engravidar, devido à preocupação por causa do surto do vírus Zika?

0 ( ) Não

1 ( ) Sim

47. Você recebeu orientação de algum profissional de saúde para evitar engravidar ou adiar a gravidez por causa do surto do vírus Zika?

0 ( ) Não

1 ( ) Sim

48. Você sabe que o virus Zika pode ser transmitido na relação sexual?

0 ( ) Não

1 ( ) Sim

49. Você recebeu orientação de algum profissional de saúde para usar o preservativo masculino (camisinha) por causa do surto do vírus Zika?

0 ( ) Não

1 ( ) Sim

50. O surto do vírus Zika fez você mudar, de alguma forma, o uso de métodos contraceptivos?

0 ( ) Não [pular para a questão 53, caso a mulher esteja grávida; caso não esteja grávida, entrevista termina aqui]

1 ( ) Sim, você começou a usar um método contraceptivo por causa do surto do vírus Zika.

2 ( ) Sim, você trocou de método contraceptivo devido ao surto do vírus Zika.

51. Qual método você começou a usar? Apenas para quem respondeu 1 e 2 na pergunta anterior

1 ( ) Pílula oral
2 ( ) Hormonal injetável
3 ( ) Preservativo masculino
4 ( ) Preservativo feminino
5 ( ) DIU
6 ( ) Diafragma
7 ( ) Tabelinha
8 ( ) Coito interrompido
9 ( ) Implante
10 ( ) Adesivo
11 ( ) Anel vaginal
12 ( ) Outros
Cite qual ou quais__________

52. Qual método você usava? Apenas para quem respondeu 1 e 2 na pergunta 72
0 ( ) Nenhum
1 ( ) Pílula oral
2 ( ) Hormonal injetável
3 ( ) Preservativo masculino
4 ( ) Preservativo feminino
5 ( ) DIU
6 ( ) Diafragma
7 ( ) Tabelinha
8 ( ) Coito interrompido
9 ( ) Implante
10 ( ) Adesivo
11 ( ) Anel vaginal
12 ( ) Outros
Cite qual ou quais__________

Somente para grávidas:
53. Você passou a usar o preservativo (camisinha) devido ao surto do vírus Zika?
0 ( ) Não
1 ( ) Sim

Questionnaire (translated into English)

Identification:
Number of the participant: __ __ __ __
Date of interview: __/__/__
Primary Health Care Service: [options]

Sociodemographic characteristics:
1. What is your date of birth? ___/___/___ [day/month/year]

2. What was the last grade you concluded at school? __ __ years of schooling

3. What is your skin color/ethinicity? [estimulated]
    1 ( ) White
    2 ( ) Brown
    3 ( ) Black
    4 ( ) Yellow (Asian)
    5 ( ) Indigenous

4. Do you work paid jobs?
    0 ( ) No
    1 ( ) Yes

5. Are you currently looking for a job?
    0 ( ) No
    1 ( ) Yes

6. Do you have private health insurance?
    0 ( ) No
    1 ( ) Yes

7. Are you currently married or in a union?
    0 ( ) No
    1 ( ) Yes

Economic characteristics:

I will now ask you a few questions about items in your household, for economic classification purposes. All electrical-electronic items I will mention must be in working condition, including any items that are stored. In case an item is not working, please, include it only if you intend to have it fixed or replaced within the next six months.

Let's get started? In your household, there is(are) ______ (READ EACH ITEM)

8. Number of passenger cars exclusively for personal (i.e., not professional) use
    0 ( ) None
    1 ( ) 1
    2 ( ) 2
    3 ( ) 3
    4 ( ) 4 and more

9. Number of monthly servants, including only those who work at least five days a week

0 ( ) None
1 ( ) 1
2 ( ) 2
3 ( ) 3
4 ( ) 4 and more

10. Number of washing machines, excluding tub washing machines
0 ( ) None
1 ( ) 1
2 ( ) 2
3 ( ) 3
4 ( ) 4 and more

11. Number of bathrooms, restrooms, powder rooms
0 ( ) None
1 ( ) 1
2 ( ) 2
3 ( ) 3
4 ( ) 4 and more

12. DVD Players, including any device that plays DVDs, and excluding Car DVD Players
0 ( ) None
1 ( ) 1
2 ( ) 2
3 ( ) 3
4 ( ) 4 and more

13. Number of refrigerators
0 ( ) None
1 ( ) 1
2 ( ) 2
3 ( ) 3
4 ( ) 4 and more

14. Number of stand-alone freezers, or freezers in two-door refrigerators
0 ( ) None
1 ( ) 1
2 ( ) 2
3 ( ) 3
4 ( ) 4 and more

15. Number of personal computers, including desktop computers, laptops, notebooks and netbooks, and excluding tablets, palmtops or smartphones
0 ( ) None
1 ( ) 1
2 ( ) 2
3 ( ) 3
4 ( ) 4 and more

16. Number of dishwashers
0 ( ) None
1 ( ) 1
2 ( ) 2
3 ( ) 3
4 ( ) 4 and more

17. Number of microwave ovens
0 ( ) None
1 ( ) 1
2 ( ) 2
3 ( ) 3
4 ( ) 4 and more

18. Number of motorcycles, not considering those used exclusively for professional activities
0 ( ) None
1 ( ) 1
2 ( ) 2
3 ( ) 3
4 ( ) 4 and more

19. Number of clothes dryers, including washers-and-dryers
0 ( ) None
1 ( ) 1
2 ( ) 2
3 ( ) 3
4 ( ) 4 and more

20. What is the householder's education? Consider the "householder" to be the person making the greatest contribution to the household income.
1 ( ) No schooling / Incomplete Elementary School
2 ( ) Elementary School Diploma / Incomplete Junior High School
3 ( ) Junior High School Diploma / Incomplete High School
4 ( ) High School Diploma / Incomplete Higher Education
5 ( ) Higher Education Degree

21. Do you – or anyone from your household – receive any social benefits?
0 ( ) No
1 ( ) Unemployment insurance
2 ( ) Direct cashier transfer
3 ( ) School cashier transfer
4 ( ) Pension
5 ( ) Retirement
6 ( ) Other

22. The water used in this household comes from...
4 ( ) The utility company's distribution system
0 ( ) Well or spring
0 ( ) Other

23. Considering the stretch of street your household is at, you would say your street is...
2 ( ) Asphalt/Paved
0 ( ) Dirt/Gravel

Now, we need some information about your sexual and contraceptive practices, so we can better understand reproductive health issues.

24. How old were you when you had your first sexual relation? __ __

25. How many sexual partners have you had in your life? __ __

26. Are you currently pregnant?
0 ( ) No
1 ( ) Yes [go to 28]
2 ( ) Doesn`t know

27. Have you ever become pregnant?
0 ( ) No [go to 31]
1 ( ) Yes

28. How many times have you ever become pregnant?__ __

29. Have you ever had an abortion?
0 ( ) No
1 ( ) Yes

30. How many children do you have? __ __

31. What is your intention on having children? [estimulated]
1 ( ) She wants to have children in the next two years
2 ( ) She wants to have children in the future
3 ( ) She does not want (more) children
4 ( ) Doesn`t know

Contraception:

32. Are you currently using contraceptive method to avoid becoming pregnant?
0 ( ) No [go to 35]
1 ( ) Yes

33. What contraceptive are you currently using now? [tick the most eficiente if she is refers more than one method]
1 ( ) Pill
2 ( ) Injectable
3 ( ) Male condom
4 ( ) Female condom
5 ( ) IUD
6 ( ) Diafragm
7 ( ) Rythm
8 ( ) Withdrawal
9 ( ) Emergency contraception pill
10 ( ) Implant
11 ( ) Patch
12 ( ) Vaginal ring
13 ( ) Female sterilization [if more than 5 years, interview stops here]
14 ( ) Male sterilization [if more than 5 years, interview stops here]
15 ( ) Other ___________________.

34. [In case the women refers more than one contraceptive, tick the other contraceptive here]
1 ( ) Pill
2 ( ) Injectable
3 ( ) Male condom
4 ( ) Female condom
5 ( ) IUD

6 ( ) Diafragm
7 ( ) Rythm
8 ( ) Withdrawal
9 ( ) Emergency contraception pill
10 ( ) Implant
11 ( ) Patch
12 ( ) Vaginal ring

Now, we are going to ask you about emergency contraception, so-called morning after pill.

35. Have you ever used emergency contraception?
0 ( ) No [go to 40]
1 ( ) Yes

36. How many times have you used emergency contraception? __ __

37. In the last year, how many times have you used emergency contraception? __ __

38. Considering the last time you used emergency contraception, were you using another contraceptive method?
0 ( ) No [go to 39]
1 ( ) Yes

38-1. If yes, which method? [Tick the most efficient]
1 ( ) Pill
2 ( ) Injectable
3 ( ) Male condom
4 ( ) Female condom
5 ( ) IUD
6 ( ) Diafragm
7 ( ) Rythm
8 ( ) Withdrawal
9 ( ) Implant
10 ( ) Patch
11 ( ) Vaginal ring
12 ( ) Other

39. Considering the last time you used emergency contraception, were you counselled by any health professional about it?
0 ( ) No
1 ( ) Yes

Now were going to ask you about Zika virus

40. Have you ever heard about Zika virus?
0 ( ) No
1 ( ) Yes

41. Are you aware that Zika vírus is trelated to microcephaly in babies??
0 ( ) No
1 ( ) Yes

In the end of 2015 (last year), there was a Zika virus outbreak, which persists so far, with cases of microcephaly confirmed in the whole country. Because of this, many women and couples decided to postpone a pregnancy. Some others think this is not necessary. What about you?

42. Do you think that the Zika virus is influencing your plans to become pregnant?
0 ( ) No
1 ( ) Yes

43. When would you like to become pregnant?
0 ( ) Immediately [go to 46]
1 ( ) In the future [go to 44]
2 ( ) Never (more) [go to 46]
3 ( ) Not sure [go to 45]

44. Do you think that Zika virus outbreak influenced your intention of becoming pregnant in the future?
0 ( ) No [go to 46]
1 ( ) Yes [go to 46]

45. Do you think that Zika virus outbreak influenced your uncertainty about becoming pregnant?
0 ( ) No
1 ( ) Yes

46. Has any health professional ever asked you about your reproductive intention because of concerns related to Zika virus outbreak?
0 ( ) No
1 ( ) Yes

47. Has any health professional ever advised you to avoid getting pregnant or postpone a pregnancy because of Zika virus outbreak?
0 ( ) No
1 ( ) Yes

48. Do you know that Zika virus can be transmitted during sexual intercourse?
0 ( ) No
1 ( ) Yes

49. Has any health professional ever advised you to use condoms because of Zika virus outbreak?

0 ( ) No
1 ( ) Yes

50. Do you think that Zika virus has somehow influenced your contraceptive method use?

0 ( ) No [go to 53, if pregnant; if not, interview ends here]
1 ( ) Yes, she started to use a method because of Zika vírus outbreak.
2 ( ) Yes, she switched the method because of Zika vírus outbreak.

51. What method have you started to use? Just for those who answered 1 and 2 in the last question
1 ( ) Pill
2 ( ) Injectable
3 ( ) Male condom
4 ( ) Female condom
5 ( ) IUD
6 ( ) Diafragm
7 ( ) Rythm
8 ( ) Withdrawal

9 ( ) Emergency contraception pill
10 ( ) Implant
11 ( ) Patch
12 ( ) Vaginal ring

52. What method were you using? Just for those who answered 1 and 2 in the last question
1 ( ) Pill
2 ( ) Injectable
3 ( ) Male condom
4 ( ) Female condom
5 ( ) IUD
6 ( ) Diafragm
7 ( ) Rythm
8 ( ) Withdrawal
9 ( ) Emergency contraception pill
10 ( ) Implant
11 ( ) Patch
12 ( ) Vaginal ring

Just for pregnant women:
53. Have you started to use condoms because of Zika virus outbreak?
0 ( ) No
1 ( ) Yes

d)

e) Has any health professional ever asked you about your reproductive intention because of concerns related to Zika virus outbreak?

f) Has any health professional ever advised you to avoid getting pregnant because of Zika virus outbreak?

g)

h)

i) Just for pregnant women: Have you started to use condoms because of Zika virus outbreak?
